# Frequent hospital presenters’ use of health information during COVID-19: results of a cross-sectional survey

**DOI:** 10.1186/s12913-023-09504-6

**Published:** 2023-06-12

**Authors:** Rebecca L. Jessup, Cassandra Bramston, Polina Putrik, Cilla Haywood, Mark Tacey, Beverley Copnell, Natali Cvetanovska, Yingting Cao, Anthony Gust, Donald Campbell, Brian Oldenburg, Hala Mehdi, Michael Kirk, Emiliano Zucchi, Adam I. Semciw, Alison Beauchamp

**Affiliations:** 1 Staying Well and Hospital Without Walls Program, Northern Health, Epping, Australia; 2 School of Allied Health, Human Services and Sport, La Trobe University, Bundoora, Australia; 3 School of Rural Health, Monash University, Warragul, Australia; 4 Department of Family Medicine, Care and Public Health Research Institute, Maastrich University, Maastricht, Netherlands; 5 Department of Medicine, University of Melbourne, Parkville, Australia; 6 Office of Research, Northern Health, Epping, Australia; 7 School of Nursing and Midwifery, La Trobe University, Bundoora, Australia; 8 Digital Health, Northern Health, Epping, Australia; 9 Academic and Research Collaborative in Health, LaTrobe University, Melbourne, Bundoora, Australia; 10 Baker Heart and Diabetes Institute, Prahran, Australia; 11 Division of Medicine, Rockhampton Hospital, Rockhampton, Australia; 12 Ethnic Communities’ Council of Victoria, Coburg, Australia; 13 School of Rural Health, Monash University, Clayton, Australia; 14 Australian Institute for Musculoskeletal Science (AIMSS), The University of Melbourne and Western Health, Melbourne, Australia

**Keywords:** COVID-19, Misinformation, Infodemic, Health communication, Health literacy

## Abstract

**Background:**

High-frequency hospital users often present with chronic and complex health conditions and are at increased risk of serious morbidity and mortality if they contract COVID-19. Understanding where high-frequency hospital users are sourcing their information, whether they understand what they find, and how they apply the information to prevent the spread of COVID-19 is essential for health authorities to be able to target communication approaches.

**Methods:**

Cross-sectional survey of 200 frequent hospital users (115 with limited English proficiency) informed by the WHO’s “Rapid, simple, flexible behavioral insights on COVID-19”. Outcome measures were source of, and trust in information, and knowledge of symptoms, preventive strategies, restrictions, and identification of misinformation.

**Results:**

The most frequently cited source of information was television (*n* = 144, 72%) followed by the internet (*n* = 84, 42%). One in four television users sought their information from overseas news outlets from their country of origin, while for those using the internet, 56% relied on Facebook and other forms of social media including YouTube and WeChat. Overall, 41.2% of those surveyed had inadequate knowledge about symptoms, 35.8% had inadequate knowledge about preventative strategies, 30.2% had inadequate knowledge about government-imposed restrictions, and 69% believed in misinformation. Half of the respondents (50%) trusted all information, and only one in five (20%) were uncertain or untrusting. English-speaking participants were almost three times more likely to have adequate knowledge about symptoms (OR 2.69, 95%CI 1.47;4.91) and imposed restrictions (OR 2.10 95%CI 1.06; 4.19), and 11 times more likely to recognize misinformation (OR 11.52 95%CI 5.39; 24.60) than those with limited English.

**Conclusion:**

Within this population of high-frequency hospital users with complex and chronic conditions, many were sourcing their information from less trustworthy or locally relevant sources, including social media and overseas news outlets. Despite this, at least half were trusting all the information that they found. Speaking a language other than English was a much greater risk factor for having inadequate knowledge about COVID-19 and believing in misinformation. Health authorities must look for methods to engage diverse communities, and tailor health messaging and education in order to reduce disparities in health outcomes.

**Supplementary Information:**

The online version contains supplementary material available at 10.1186/s12913-023-09504-6.

## Introduction

Successful public health responses to pandemics require individuals to have sufficient ability to access, clearly understand and easily apply relevant information about symptoms, prevention, testing and containment strategies. Populations with adequate levels of health literacy, defined as the ability to find, access and use information in order to promote and maintain health [1], are essential to the of success public health responses to COVID-19. Population studies in the USA, Canada and Australia estimate the prevalence of inadequate health literacy ranges from 36 to 60% [2–4]. This means that many individuals face significant challenges in accessing, understanding and applying trustworthy health information about COVID-19 [5].

Studies performed during the COVID-19 pandemic across the world have demonstrated that a variety of social and demographic factors are associated with how and where individuals have sought relevant information. These factors have included socioeconomic background, age, education, health literacy and even personal politics [6–9]. Studies have also demonstrated that where individuals seek information and how much they trust this information has an influence on their prevention intent [10–13].

Individuals with chronic and complex care needs who are dependent upon regular hospital care are at increased risk of serious morbidity and mortality if they contract COVID-19 [14, 15]. There has been much speculation over the past two years about the indirect contribution of the pandemic on excess mortality rates related to reductions in hospital attendance, with presentations for potentially life-threatening conditions, such as myocardial infarction and stroke, declining by as much as 48% [16, 17]. Frequent hospital users, defined as those who have had three or more hospital admissions in the previous 12 months, are one group that has been shown to have dramatically reduced their emergency attendance [18] and this may have contributed to delays in necessary care for people with chronic and complex care needs.

Understanding where high-frequency hospital users with chronic and complex health needs might be sourcing their health information, whether they understand what they find, and how they apply the information to prevent the spread of COVID-19 is essential for health authorities to be able to target communication approaches to ensure that those requiring care continue to seek help in a timely way. The aim of this research is therefore to determine where frequent hospital users from a diverse range of cultural and linguistic backgrounds have accessed information during COVID-19, whether they trusted the information they found, and how they have interpreted that information to support their health.

## Methods

### Theoretical models

When designing this study, we grounded the work in Longo’s integrated model of health information seeking behaviors [19] and the Risk Information Seeking and Processing (RISP) model [20]. Longo’s model posits that health seeking behaviours are influenced by both the contextual (health, social support, environment, health care structures) and the personal (socioeconomic position, culture, language, attitudes, behaviours). The RISP model posits that an important trigger for health seeking information is the level of worry that is aroused by a perceived risk; an important additional motivator for health seeking behavior in the context of stress and fear responses to pandemic. These models propose that individuals will be both passive and active receivers of information, but that the seeking out of information will only occur when there is a perceived gap between a person’s knowledge and the reality they are faced with.

### Design, setting and sample

In Australia, the largest outbreak of COVID-19 in 2020 was in Melbourne, accounting for 75% of all Australian cases (*n* = 20,345 on 4th of December), and 90% of all deaths (*n* = 820). In response to rising COVID-19 case numbers, stage 3 restrictions were imposed on the 8th of July 2020 which was extended to stage 4 restrictions on 2nd of August. Stage 3 restrictions saw the closure of non-essential shops, and required residents to stay at home with only four exceptions: shopping for food and essential items, care and caregiving, daily exercise, work and study if unable to do it at home. In addition to restrictions in Stage 3, Stage 4 imposed a 5 km radius of travel (except for seeking medical care or attending essential work), allowed only one person per household to leave to shop, and exercise was limited to one hour per day. Cases in Victoria peaked on the 5th of August 2020, when 725 new cases were reported in the State over a 24-h period. This study was conducted from the 6th of July to the 24th of August 2020 during the peak of the 2020 pandemic in Melbourne. Importantly, it is the same population that was impacted by rising cases in 2021, with approximately 75% of all cases located in Melbourne’s north as we approached the peak of the wave in October 2021.

Northern Health (NH) is the major provider of hospital care in Melbourne’s north. Residents living in the area are culturally and linguistically diverse, speaking more than 100 languages. The area has lower levels of income, educational attainment and health literacy and higher rates of unemployment than Victorian state averages [21–23]. The catchment also has the highest population of recently arrived refugees in Victoria, most originating from the Middle East [24]. The catchment covers about 10% of Victoria’s population, however a third of Victoria’s COVID-19 cases were located in this area at the peak of the pandemic in 2020, increasing to almost 70% of all cases during 2021 [25].

We conducted a cross-sectional survey by phone with a sample of Australian-born patients and patients with limited-English proficiency from migrant and refugee backgrounds. All patients had complex and chronic conditions, and a history of frequent hospital care at NH. We selected our study sample from the top 5000 most frequent users of inpatient care identified by a case-finding algorithm developed by the Victorian Department of Health and Human Services [26]. The algorithm uses the following risk factors (criteria) to predict a future risk of hospitalization over the next 12 months; age, number of unplanned admissions in the past 6 months, number of emergency department visits in the past 3 months, hospital stay caused by selected progressive conditions and co-morbidities (such as asthma, kidney disease, COPD, heart disease, rheumatoid arthritis), smoking status, and place of residence (aged care or private residence). Each criterion provides a weighted value risk of future admissions and is triggered once a points threshold is reached. The model has been found to accurately identify patients who will be admitted three or more times in the following 12 months 32% of the time [26]. The patients in this study were all identified as at risk of future admissions and were considered to have the highest overall complexity of all patients attending NH for treatment. The study focused on how this population accessed, interpreted and whether they trusted information specifically related to COVID-19 and not their other health conditions.

### Measures

Outcome measures included sources of information, perceived trustworthiness of information knowledge of symptoms, preventive strategies, government restrictions, and identification of misinformation. To measure these outcomes we adapted the World Health Organization (WHO) “Rapid, simple, flexible behavioral insights on COVID-19” survey with additional researcher generated questions based on the Longo and RISP frameworks created to collect data relevant to the local context [27] (Supplementary File 1). The WHO survey was developed from validated instruments to monitor knowledge, risk perceptions, preventive behavior and trust in populations to inform pandemic outbreak response [27].

Sources of information were measured using the question ‘tell me about where you have looked for information since the COVID-19 pandemic began?’. Respondents were provided eight response options (internet, television, radio, newspaper, family/friends, religious groups, general practitioner/ health professional, other). Participants could choose more than one source. They were then asked to elaborate on their response to provide specific sources in an open response format (e.g., if using the internet, was the source government websites, social media sites, etc.).

To understand trust in information, we asked participants the open-ended question “how much do you trust the information you are finding about COVID-19”. Initially this question was intended for a comprehensive thematic analysis, however many of the answers were provided as simple one or two-word responses (e.g. mostly or 100%) or short sentences (I’m not sure what to trust) that fitted better with a simple content analysis. To this end, categorized responses to pre-formulated coding rules as “believes or trusts everything”, “partially believes and this may depend on the source”, “don’t know if I can trust” and “don’t trust anything”. Both questions about source of information and trustworthiness of information were created grounded in the theoretical models and were developed by RLJ and reviewed by AB who have expertise in health literacy. They were additionallytrialed by two consumers with lived experience. No adjustments were required following the trial.

Knowledge of symptoms was measured using the WHO survey 10 response (True/False and don’t know) items, to the question ‘which of the following can be symptoms of COVID-19?’. We defined adequate knowledge of symptoms as knowledge of all three common COVID-19 symptoms as identified by the WHO (sore throat, dry cough and fever) [28]. ‘Don’t know’ responses were considered inadequate knowledge and were coded along with incorrect responses for these items.

Knowledge of preventive strategies was assessed using the WHO survey 15 response (True/False and don’t know) items in response to the question ‘which of the following are effective measures to prevent the spread of COVID-19?’. We defined adequate knowledge of preventive strategies as able to identify at least 80% of the 15 strategies on the WHO survey correctly (79% and below was considered inadequate knowledge of preventive strategies). This cut point was arbitrary, but was premised on the fact that preventive strategies were highly publicised and would be close to 100% understood by anyone with access to trustworthy sources of information. ‘Don’t know’ responses were considered inadequate knowledge and were coded as incorrect responses for these items. In addition, we looked at knowledge of misinformation about preventing COVID-19 and defined this as correctly recognizing misinformation around preventative strategies including use of garlic, ginger or lemon to prevent COVID-19, antibiotics, influenza vaccine, disinfecting postage, herbal remedies.

Knowledge of government restrictions were assessed using a researcher generated question (RLJ and reviewed by AB) that was trialed by two consumers. There were four exceptions to staying home that applied during stage 3 and stage 4 restrictions. These were; attending essential employment, seeking or providing care, exercising, and purchasing groceries or pharmaceuticals. Information about restrictions was widely publicized, however many of our sample were not working and so the work-related exception did not apply. We therefore considered adequate knowledge as being able to name three of four exemptions for leaving home.

Sociodemographic variables collected through self-report included age, gender, level of educational attainment, employment status, country of birth, primary language spoken at home, living arrangements, and household income.

### Survey administration procedure

The survey was administered by phone, and all participants provided verbal consent to participate as per the ethics protocol. Stratified random sampling was used to select patients across English and the top 10 other language groups in the region (Arabic, Italian, Assyrian/ Chaldean, Turkish, Greek, Macedonian, Mandarin, Persian, Vietnamese, Hindi/ Punjabi), age, and gender. If a patient declined participation, the next patient on the random sample list was approached, until 200 participants were recruited. 200 participants was chosen as the sample size based on resources available to conduct the survey. We aimed to recruit a minimum 50% with limited English proficiency. Patients were excluded if they were unable to provide informed consent or spoke a language other than English or those in the top 10. Interpreters were made available for all individuals where English was not the preferred language.

### Ethical approval

This project was approved by the Northern Health Human Research Ethics Committee (LNR 64196). The committee included consumer representatives who provided feedback on the survey questions and on study methods.

### Statistical analysis

Data on sources of information were reported as proportions for each category. Content analysis was conducted on open ended responses to the question ‘how much do you trust the information you have been reading and hearing?’ and responses were categorized across the following pre-formulated codes: 1) I believe everything, 2) I partially believe / am somewhat unsure/ depends on the source of the information 3) I don’t know if I trust the information, I am uncertain 4) I don’t trust anything. Two researchers (CH and BC) independently applied the coding. We aimed to have 100% agreement between researchers so that a final proportion could be applied to each category, so where differences in coding occurred, these were discussed until agreement was found or a third researcher (RLJ) was consulted.

We used logistic regression to explore the associations between age (≥ 65 vs. < 65 years), gender (female vs. male), primary language (English vs. other), living alone (vs. with others), education (completed vs. did not complete high school), and different knowledge-related outcomes, which included knowledge of symptoms, knowledge of preventive strategies, knowledge of government restrictions, and knowledge of misinformation. Univariate models were conducted first followed by multivariate models, with the latter including only those variables found to be significant at *p*-value ≤ 0.05 in univariate models. Variables that were not added to final model based on results of univariable analyses were further tested in a sensitivity analysis. These variables were added to the final model one by one to explore possible confounding effects. Odds ratios (OR) are presented with 95% Confidence Intervals (CI). Data were analyzed using Stata version 15.

## Results

A total of 272 patients were invited to participate before a sample size of 200 was achieved (response rate 74%). Mean age was 66.5 years (SD 15.6, range 22–99 years), with 97 (49%) respondents female. More than two-thirds of participants did not complete high school, and most were on very low incomes. Only 13 (7%) participants were currently working. Study participants were culturally diverse, with 115 (58%) speaking a primary language other than English (surveyed using interpreters) and 152 (76%) born overseas. Over one-third of participants (*n* = 74) lived in multi-generation households (Table 1). No participants reported having contracted COVID-19 prior to, or at the time of, participation had COVID-19 (nor had any member of their household).

**Table 1:** Descriptive characteristics of sample (*n* = 200). Data are shown as *n* (%) unless otherwise specified

Mean age in year (SD)	66.5 (15.6)
Age group, years	
18–40	14 (7.0)
41–55	36 (18.0)
56—70	62 (15.6)
71–98	88 (22.1)
Gender	
Female	97 (48.5)
Male	103 (51.5)
Highest level of education	
Did not complete high school	135 (67.50)
Completed high school	25 (12.5)
Trade certificate/ Technical and further education (TAFE) qualification	14 (7.0)
Diploma or bachelor degree	19 (9.5)
Masters or doctoral degree	6 (3.0)
Household income	
< $35,000	163 (81.5)
35,000—50,000	9 (4.5)
> $50,000	12 (6.0)
Rather not say	16 (8.0)
Living arrangements	
Lives alone	33 (16.5)
Lives with partner	23 (11.5)
Lives with partner and children	61 (30.5)
Lives in multi-generation household^a^	74 (37)
Lives with others (flatmates)	6 (3.0)
Other	3 (1.5)
Born in Australia	48 (24)
Primary language	
Arabic	26 (13)
Assyrian Neo-Aramaic/ Chaldean Neo-Aramaic	9 (4.5)
English	85 (42.5)
Greek	16 (8.0)
Italian	19 (9.5)
Macedonian	7 (3.5)
Mandarin	5 (2.5)
Persian (excluding Dari)	7 (3.5)
Punjabi / Hindi	7 (3.5)
Turkish	12 (6.0)
Vietnamese	7 (3.5)
Currently working	16 (8.0)
Inadequate knowledge^b^	
Symptoms (*n* = 194)	80 (41.2)
Prevention (*n* = 190)	68 (35.8)
Stage 3 or 4 local restrictions (*n* = 182)	55 (30.2)
Misinformation (*n* = 190)	131 (69.0)

^a^Defined as adults with one or more children, living with ageing parents or grandchildren

^b^Inadequate knowledge defined as follows: Symptoms = unable to identify all three common symptoms (sore throat, fever, dry cough); Prevention = recognizes 79% or less preventive strategies correctly; Stage 3 or 4 local restrictions = unable to name 2 out of 3 local restrictions (excluding work-related reasons; only includes people not currently working); Misinformation = states that 3 or more of garlic, antibiotics, Fluvax, disinfecting postage, or herbal remedies are preventive strategies

**Fig. 1:**
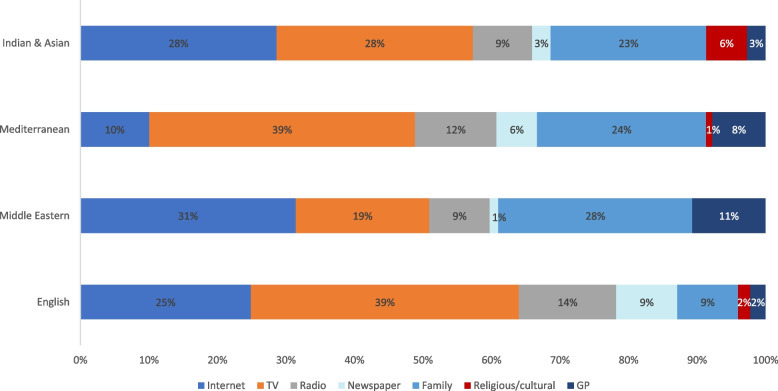
Information sources by language group. * *n* = 200. Data shown as individuals. *Values have been rounded to the nearest whole number

**Fig. 2:**
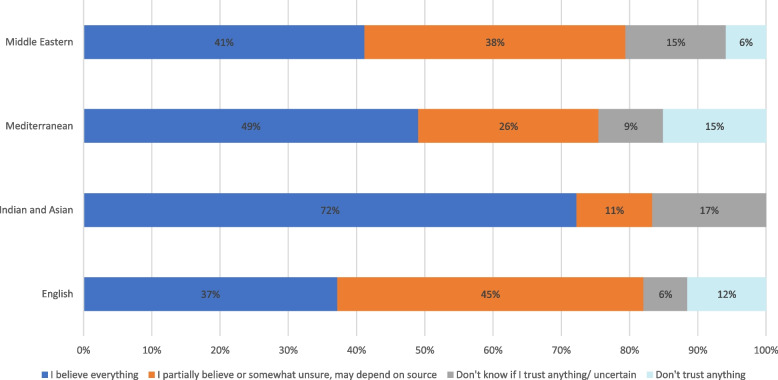
Trust in information by language group. * *n* = 200. Data shown as percentage. *Values have been rounded to the nearest whole number

Figures 1 and 2 provide an overview of information sources and perceived trustworthiness according to primary language spoken. The most frequently cited source of information was television (*n* = 144, 72%) followed by the internet (*n* = 84, 42%). Sources in the ‘other’ category included work colleagues (3 individuals) and leaflets dropped in the mailbox (1 individual). Content analysis of the free text responses of specific sources of information found that for those sourcing their information on the television, 83 (58%) watched free to air privately owned television stations (channel 7, 9 and 10), while 36 (25%) sought their information from overseas news outlets from their country of origin. For those using the internet, 48 (56%) relied on Facebook and other forms of social media including YouTube and WeChat. In terms of trustworthiness of the information, 45% of respondents said they trust everything, 34% reported partially trusting information depending on the source, and 19% said they were uncertain about the information or that they didn’t trust anything. Those who were surveyed in either Punjabi, Hindi, Vietnamese or Chinese languages were the most trusting of information with 72% identifying that they believe everything (Fig. 2).

Overall, 41.2% of this population had inadequate symptom knowledge, 35.8% had inadequate preventive strategy knowledge, 30.2% had inadequate knowledge of restrictions, and 69% were not able to adequately recognize misinformation (Table 1). Table 2 presents demographic and other predictors of adequate symptom knowledge, preventive strategies, local restrictions and misinformation. In univariate logistic regression, being aged ≥ 65 years was associated with less ability to recognize misinformation (OR 0.28, 95%CI 0.28;0.97), compared to younger participants. Having adequate English proficiency was associated with an almost 3 times greater knowledge of symptoms (OR 2.69, 95%CI 1.47; 4.91), and greater knowledge of local restrictions (OR 2.10, 95%CI 1.06; 4.19) compared to those with limited English proficiency. Participants with adequate English were also 11 times more likely to recognize misinformation about preventive strategies than those with limited English proficiency (OR 11.52, 95% CI 5.39; 24.60). Completion of secondary education was associated with greater likelihood of having adequate knowledge of symptoms (OR 2.13, 95%CI 1.12;4.07).

**Table 2:** Univariate logistic regression: association between demographic variables and adequate knowledge of symptoms, preventive strategies, local restrictions exemptions and misinformation

**Predictor**	**Adequate knowledge of symptoms (*n* = 193)**	**Adequate knowledge of preventive strategies (*n* = 190)**	**Adequate knowledge of three local restrictions (*n* = 182)**^a^	**Adequate recognition of misinformation (*n* = 190)**
	***OR 95%CI***	***OR 95%CI***	***OR 95%CI***	***OR 95%CI***
Female vs. Male	0.86 [0.48;1.52]	1.23 [0.68; 2.24]	1.27 [0.67; 2.40]	0.83 [0.45; 1.54]
Age ≥ 65 vs age < 65	0.74 [0.42; 1.32]	0.88 [0.48; 1.59]	0.60 [0.31; 1.18]	**0.52 [0.28; 0.97]**
Primary language English vs other	**2.69 [1.47; 4.91]**	0.58 [0.32; 1.05]	**2.10 [1.06; 4.19]**	**11.52 [5.39; 24.60]**
Living alone vs with others	1.42 [0.64; 3.13]	0.78 [0.36; 1.70]	0.90 [0.38; 2.14]	1.95 [0.90, 4.26]
Completed high school versus not	**2.13 [1.12; 4.07]**	0.76 [0.40; 1.42]	1.41 [0.69; 2.88]	1.66 [0.87; 3.12]

Adequate knowledge of symptoms defined as knowledge of all three common COVID-19 symptoms as identified by the World Health Organization (WHO) (sore throat, dry cough and fever)

Adequate knowledge of preventive strategies defined as able to identify at least 80% of the 15 strategies on the WHO survey correctly

Adequate knowledge of local restrictions defined as being able to correctly name 2 out of 3 exemptions for leaving home (excluding work-related reasons)

Adequate recognition of misinformation defined as reports ≤ 2 of garlic, antibiotics, Fluvax, disinfecting postage, or herbal remedies as being preventive strategies

^a^Conducted only in participants who are not working or who are retired

The association seen between proficient English and greater knowledge of symptoms and three of the four local restrictions remained significant after inclusion of education in the model (adjusted OR for symptoms 2.64, 95%CI 1.42; 4.89, for restrictions 2.1 95%CI 1.06, 4.19), and for recognition of misinformation after inclusion of age in the model (adjusted OR 11.55, 95%CI 5.36, 24.87) (Table 3). Sensitivity analyses confirmed no important confounding effects were missed (data not shown).

**Table 3:** Multivariate logistic regression: association between demographic variables and adequate knowledge of symptoms, preventive strategies, local restrictions exemptions, and misinformation

**Predictor**	**Adequate knowledge of symptoms** **(*n* = 193)**^a^	**Adequate knowledge of preventive strategies** **(*n* = 190)**	**Adequate knowledge of three local restrictions** **(*n* = 182)**^b^	**Adequate recognition of misinformation** **(*n* = 190)**^c^
	***OR 95%CI***	***OR 95%CI***	***OR 95%CI***	***OR 95%CI***
Female vs. Male	-	-	-	-
Age ≥ 65 vs. age < 65	-	-	-	0.51 [0.25, 1.06]
Primary language English vs. other	**2.64 [1.42; 4.89]**	-	**2.10 [1.06; 4.19]**	**11.55 [5.36, 24.87]**
Living alone vs. with others	-	-	-	-
Completed high school vs. not	1.92 [0.99; 3.74]	-	-	-

Adequate knowledge of symptoms defined as knowledge of all three common COVID-19 symptoms as identified by the World Health Organization (WHO) (sore throat, dry cough and fever)

Adequate knowledge of preventive strategies defined as able to identify at least 80% of the 15 strategies on the WHO survey correctly

Adequate knowledge of local restrictions defined as being able to correctly name 2 out of 3 exemptions for leaving home (excluding work-related reasons)

Adequate recognition of misinformation defined as reports ≤ 2 of garlic, antibiotics, Fluvax, disinfecting postage, or herbal remedies as being preventive strategies

^a^Only primary language and completed high school included in the model

^b^Only primary language included in the model

^c^Only age and primary language included in the model

## Discussion

Amongst high frequency hospital users, we identified high rates of inadequate knowledge of symptoms, preventive strategies and restrictions, particularly from participants with limited English proficiency. Those with limited English were also 11 times more likely than those with adequate English to believe misinformation around preventive strategies for COVID-19. There was a high level of uncertainty and mistrust of information which varied across language groups. We found that even when adjusted for age and education, speaking a primary language other than English was associated with having less knowledge of COVID-19 symptoms compared to participants who spoke English. Being aged 65 years and over was associated with having less knowledge of misinformation compared to younger participants.

Access to timely and accurate information from a trustworthy source is essential during a pandemic. Good communication strategies can alleviate population fears and dispel misinformation and disinformation that may lead to adoption of practices which lack an evidence base and put individuals and communities at risk [29]. Health and risk communication theories have long recognized the importance of testing messages with diverse groups of people to ensure that the information has been translated in the way that it was intended [29]. Provision of timely, accurate and culturally appropriate translated materials on COVID-19 was a challenge during the first and second pandemic waves in Melbourne [25]. Information was aimed at early detection, prevention, and containment of further spread of the virus – this changed daily and there was little opportunity to check that messages were understood accurately by all intended audiences.

Similar to many countries globally, migrant communities in Australia were disproportionately impacted by COVID-19, with the death rate for those born overseas more than 3 times that of those born in Australia [30]. In August 2020, the Refugee Council of Australia identified that both national and state government-translated coronavirus information was ‘nonsensical’, that the information was not being kept up to date, or that it was not culturally appropriate [31]. In addition, some key advocates for the anti-vaccination narrative may have specifically targeted migrant communities [32]. Our findings that the most common source of information was television, followed by the internet, was similar to findings overseas [33, 34]. However, unlike these studies which found that the most common source of online information was government websites, our participants relied heavily on information provided through social media. Social media is the primary source of the misinformation infodemic [35] and it is possible that inadequate dispelling of this misinformation by trustworthy sources may have contributed to lower rates of vaccination and higher mortality rates.

Approximately 20% of the population surveyed were newly arrived migrants and refugees originating from the Middle East. In 2021, this population experienced devastating outcomes from COVID-19, with a mortality rate of 29.3 deaths per 100,000 people – more than 13 times higher than the rate for Australian born citizens (2.3 COVID-19 deaths per 100,000) [30]. Along with being disproportionally at risk of contracting COVID-19, refugees and asylum seekers experience additional vulnerabilities around their ability to trust information provided by figures of authority [36]. As most trustworthy information is provided by government sources, and much of it was not accurately translated, this may have further disadvantaged these communities and placed them at greater risk of contracting and spreading the virus. Our research found that religious and cultural leaders were an important source of trusted information for many in this population, and public health authorities should consider early engagement in the future to improve reach in this community.

To keep the whole population safe and reduce the spread of infection, governments and leaders need to understand much more about what influences individual and collective behaviors. Learnings from the 2009/2010 Australian influenza epidemic demonstrated that there was not enough research available on the complex interplay of rapidly changing epidemiology, media attention, control measures, risk perception, and public health behaviors associated with pandemics [37]. The WHO survey used in this study was specifically designed to gain insight into these issues, and its application may assist to address some of these research gaps. This study has provided some new insight into relationships between demographics, knowledge, protective behaviors, perceptions, and trust in Australia. This contextually relevant knowledge on how communities are sourcing and interpreting information may help governments and health communicators to provide appropriate and equitable messaging on response measures and to address misinformation and disinformation as it emerges [38].

There are some limitations to this study. As the study was conducted in a single hospital network in Melbourne the results may not be generalizable. In addition, the surveyed cohort focused on only the top 10 most spoken languages other than English at the hospital and therefore the experience of some important groups with low representation may not have been captured. Importantly, the study was conducted in a region of Melbourne that was disproportionately impacted by COVID-19 [39], and it focuses on frequent hospital users who, to the best of our knowledge, have not been represented in the literature to date.

## Conclusion

Within a vulnerable population with complex and chronic conditions, speaking a language other than English was found to expose participants to greater risks due to inadequate knowledge. To reduce transmission, morbidity and mortality associated with COVID-19, health authorities must tailor health messaging and education to those disadvantaged communities in order to reduce disparities in health outcomes.

## Supplementary Information

**Additional file 1.** 

## Data Availability

The datasets used and/or analysed during the current study available from the corresponding author on reasonable request.

## Abbreviations

NH: Northern Health
WHO: World Health Organization
