# Supplementary material for: Frequent hospital presenters’ use of health information during COVID-19: results of a cross-sectional survey
Source: BMC Health Serv Res. 2023 Jun 12;23:616. doi: 10.1186/s12913-023-09504-6 (PMC10258478; doi:10.1186/s12913-023-09504-6)
Supplement: Supplementary file 1 — Additional file 1. [file 12913_2023_9504_MOESM1_ESM.docx]

**Supplementary File 1**

**Accessing and applying information during COVID-19**

**Patient Survey**

| **Outcome measure** | **Items** | **Analysis** |
| --- | --- | --- |
| Source of information during COVID19 | Tell me about where you have looked for information since COVID19 pandemic began?  Please choose all that apply from the following response options:  Internet  Television news broadcast  Radio news broadcast  Newspapers  Communication from family or friends  Communication from religious or cultural groups  GP/ Health professional  Other  Participants were asked to provide detail about the source next to their response. | Descriptive analysis of source of information |
| Perceived trustworthiness of information | How much do you trust the information you are finding about COVID-19? | Content analysis. Responses categorised into the following:   - I believe everything - I partially believe, or am somewhat unsure, depends on the source of the information - I don’t know if I trust the information, I am uncertain - I don’t trust anything |
| Knowledge of government restrictions | Can you name the four reasons you are allowed to leave home during stage 3 restrictions?  To shop for groceries or pharmaceuticals  To exercise  To seek healthcare or provide care  To attend essential employment | Adequate knowledge, defined as able to name three out of four exemptions |
| Knowledge of COVID19 symptoms (from WHO survey) **^1^** | Which of the following can be symptoms of the novel coronavirus?  Please select as many as apply from the following response options  [*] Yes - Related to the coronavirus  [*] No - Not related to the coronavirus  [*] Don’t know  Fever (common symptom)  Dry cough (common symptom)  Shortness of breath  Sore throat (common symptom)  Runny or stuffy nose  Muscle or body aches  Headaches  Fatigue (tiredness)  Diarrhea  Loss of taste and smell  Additional? | Adequate knowledge defined as able to identify all three common COVID-19 symptoms |
| Knowledge of preventative strategies (includes misinformation) (from WHO survey) **^1^** | I follow the recommendations from authorities in Australia to prevent spread of novel coronavirus. Not at all [*] [*] [*] [*] [*] [*] [*] Very much so  Which of the following are effective measures to prevent the spread and infection of the novel coronavirus?  Please evaluate all preventive measures listed below with the following response options:  [*] Yes  [*] No  [*] Don’t know   - Hand washing for at least 20 seconds - Avoiding touching your eyes, nose, and mouth with unwashed hands - Use of disinfectants to clean hands when soap and water is not available for washing hands - Staying home when you are sick or when you have a cold - Herbal remedies/ supplements (**misinformation**) - Covering your mouth when you cough - Using caution when opening letters/ disinfecting postage (**misinformation**) - Getting the flu vaccine (**misinformation**) - Wearing a face mask - Using antibiotics (**misinformation**) - Physical distancing (keeping minimum 2 metres between you and other persons outside your household) - Self-isolation - Disinfecting surfaces - Disinfecting the mobile phone - Eating garlic, ginger, lemon (**misinformation**) | Adequate knowledge defined as able to identify 80% of preventative strategies correctly  Knowledge of misinformation defined as correctly recognising all four misinformation items |

**References:**

1. World Health Organisation (2020). Survey Tool and Guidance: Rapid, simple, flexible behavioural insights on COVID-19. Available at <http://www.euro.who.int/en/health-topics/health-emergencies/coronavirus-covid-19/novel-coronavirus-2019-ncov-technical-guidance/who-tool-for-behavioural-insights-on-covid-19> Accessed May 2020
